# The Role of the NLRP3 Inflammasome in Mediating Glomerular and Tubular Injury in Diabetic Nephropathy

**DOI:** 10.3389/fphys.2022.907504

**Published:** 2022-06-09

**Authors:** B. M. Williams, C. L. Cliff, K. Lee, P. E. Squires, C. E. Hills

**Affiliations:** ^1^ School of Life Sciences, University of Lincoln, Lincoln, United Kingdom; ^2^ Lincoln County Hospital, Lincoln, United Kingdom

**Keywords:** inflammation, diabetic nephropathy, NLRP3, diabetes, fibrosis

## Abstract

The NOD-like receptor protein 3 (NLRP3) inflammasome is a multi-protein signalling complex integral to the chronic inflammatory response, activated in response to sterile and non-sterile cellular damage. The assembly and activation of the NLRP3 inflammasome comprise a two-step process involving nuclear factor kappa B (NFkB)-mediated priming, followed by canonical, non-canonical or alternative signalling pathways. These result in the maturation and release of inflammatory cytokines interleukin 1 beta (IL1ß) and interleukin-18 (IL18), which are associated with chronic inflammatory conditions including diabetic kidney disease. Diabetic nephropathy is a condition affecting ∼40% of people with diabetes, the key underlying pathology of which is tubulointerstitial inflammation and fibrosis. There is growing evidence to suggest the involvement of the NLRP3 inflammasome in this chronic inflammation. Early deterioration of kidney function begins in the glomerulus, with tubular inflammation dictating the progression of late-stage disease. Priming and activation of the NLRP3 inflammasome have been linked to several clinical markers of nephropathy including proteinuria and albuminuria, in addition to morphological changes including mesangial expansion. Treatment options for diabetic nephropathy are limited, and research that examines the impact of directly targeting the NLRP3 inflammasome, or associated downstream components are beginning to gain favour, with several agents currently in clinical trials. This review will explore a role for NLRP3 inflammasome activation and signalling in mediating inflammation in diabetic nephropathy, specifically in the glomerulus and proximal tubule, before briefly describing the current position of therapeutic research in this field.

## The Role of NLRP3 in Diabetes

Inflammation is the body’s protective response to injury, including trauma (Huber-Lang et al., 2018), stress (Liu et al., 2017), ischaemia (De Bhailís et al., 2021) and infection (Kaufmann et al., 2018). The NOD-like receptor protein 3 (NLRP3) inflammasome is an important mediator of the innate inflammatory response to stimuli including extracellular signals, e.g., adenosine triphosphate (ATP) and pathogens, including bacteria and fungi (Fusco et al., 2020). In the absence of infection or disease, NLRP3 activity is tightly regulated, whilst dysregulated activation is strongly associated with chronic inflammatory diseases (Chen et al., 2021), including atherosclerosis (Sharma et al., 2021) and neurodegeneration (Mondal et al., 2021). Dogma supports NLRP3-induced inflammation as fundamental to changes in glucose tolerance, insulin resistance and inflammation as observed in both type 1 (T1DM) and type 2 diabetes mellitus (T2DM) (Ding et al., 2019). Additionally, as a protein complex that bridges our innate and adaptive immune response (Tianli Zhang et al., 2021), it is integral to the underlying pathogenesis of secondary microvascular complications of diabetes, including impaired diabetic wound healing (Huang et al., 2020), diabetic retinopathy (Ge et al., 2022) and diabetic kidney disease (Ram et al., 2020; Feng et al., 2021).

Despite advances in the clinical management of diabetes, the incidence of nephropathy continues to rise (DeFronzo et al., 2021), with an estimated 40% of individuals developing renal complications at some stage of their life (Remuzzi et al., 2002; Dagar et al., 2021). Chronic conditions of inflammation, often seen with advancing age, and in co-morbidities including hypertension (Tziomalos and Athyros, 2015), coronary artery disease (Roy et al., 2021) and obesity (Qin et al., 2021), are all risk factors for the onset of kidney disease, where NLRP3 activation is pivotal to pathology (Menini et al., 2020). Therefore, targeting the NLRP3 inflammasome is of obvious therapeutic potential in the future management of both nephropathy and other age-associated morbidities.

## The NLRP3 Inflammasome

The premise of an “inflammasome” was first proposed in 2002, by Martinon *et al.*, as a caspase-activating complex that led to the production of interleukin 1 beta (IL1ß) (Martinon et al., 2002). Although several inflammasomes have since been identified, including NLRP1 and NLRP2, the NLRP3 inflammasome is the most extensively characterised to date (Zahid et al., 2019). The NLRP3 inflammasome consists of NLRP3, an “apoptosis-associated speck-like protein containing a CARD (caspase recruitment domain)” (ASC) and pro-caspase-1 (Nagar et al., 2021). NLRP3 itself is formed of 3 domains: the C-terminal leucine-rich repeat (LRR) domain, the NACHT domain and an N-terminal effector pyrin domain (PYD) (Hafner-Bratkovič et al., 2018). To activate the NLRP3 inflammasome complex, recruitment of the ASC precedes the recruitment of pro-caspase-1 through CARD/CARD interactions (Masumoto et al., 1999; Lu et al., 2014), culminating in cleavage of pro-caspase-1 and subsequent catalytic conversion of pro-interleukin-1beta (pro-IL1ß) and pro-interleukin-18 (pro-IL18) into their mature, active forms (Lu et al., 2014; El-Sharkawy et al., 2020).

Activation of NLRP3 can occur in response to microbial infection, following exposure to pathogens and pathogen-associated molecular patterns (PAMPs) such as *Mycoplasma pneumonia,* which is implicated with pneumonia and asthma (Luo et al., 2021) and *Citrobacter freundii*, which causes food poisoning and urinary tract infections (Liu et al., 2021). In the absence of infection, the NLRP3 inflammasome is a key mediator of sterile inflammation and is activated in response to injury and disease through stimuli that include glycaemic injury (Castro et al., 2021), oxidative stress (Tao et al., 2021), and the release of danger-associated molecular patterns (DAMPs), e.g., ATP (Rumora et al., 2021).

These diverse stimuli evoke a two-step process, consisting of both priming and activation (El-Rous et al., 2021). Priming is initiated in response to stimuli that include microbial components (Negash et al., 2019) and/or inflammatory cytokines, such as tumour necrosis factor-alpha (TNFα) (Franchi et al., 2009), which lead to the activation of the transcription factor nuclear factor-kappa B (NFkB) (El-Rous et al., 2021). Translocation of NFkB to the nucleus subsequently upregulates transcription of NLRP3 and pro-IL1ß (Kelley et al., 2019). Interluekin 1 beta is not constitutively expressed and is an indicator of NLPR3 inflammasome priming (Park et al., 2020). This step is regulated through a variety of mechanisms including microRNAs (miRNAs) (Tufekci et al., 2021), which inhibit the translation of NLRP3 (Haneklaus et al., 2012; Mangan et al., 2018). Once primed, PAMPs or DAMPs through their ability to bind to either the purinergic P2X7 receptor (P2X7R; DAMPS) (Yiwen Zhang et al., 2021) or toll-like receptors (TLRs; PAMPs), activate the NLRP3 inflammasome through recruitment and binding of ASC and pro-caspase-1 to NLRP3 (Mangan et al., 2018). This predominantly leads to potassium efflux (Wang and Hauenstein, 2020), but can evoke changes in intracellular calcium (Jäger et al., 2020), lysosome destabilisation (Svadlakova et al., 2020) and the production of reactive oxygen species (ROS) (Li et al., 2021).

**FIGURE 1:**
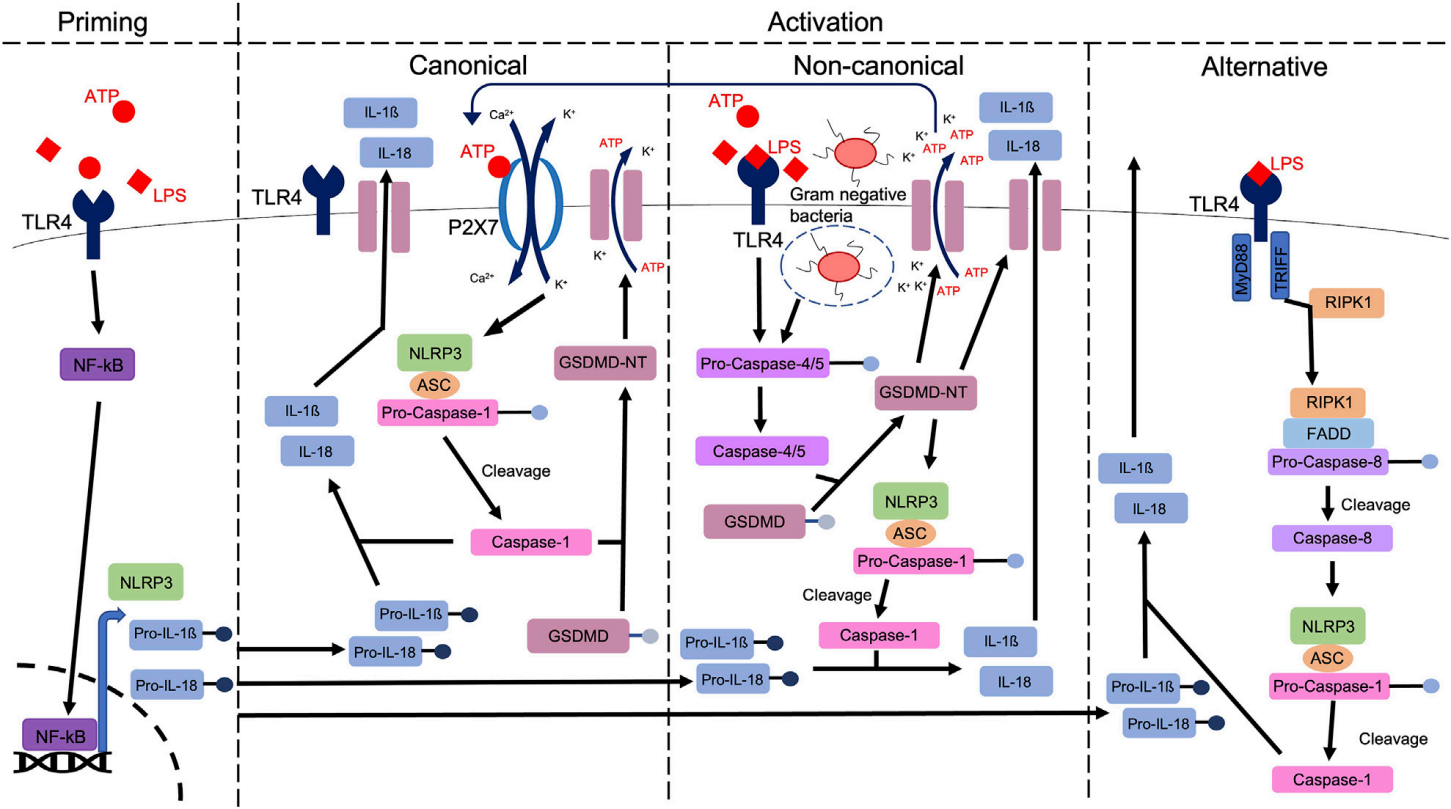
A schematic highlighting the priming and various activation pathways of the NLRP3 inflammasome. A priming signal (e.g., ATP), activates NFkB, which is translocated to the nucleus, to upregulate the expression of NLRP3, pro-IL1ß and pro-IL18. *Via* either canonical, non-canonical or alternative pathways, caspase-1 mediated cleavage of pro-IL1ß and pro-IL18 produces mature IL1ß and IL18, ahead of release from the cell, through membrane bound channels, including Gasdermin D (GSDMD) pores. In canonical signalling, stimulation of receptors including TLR4 and P2X7R evokes potassium efflux and calcium influx, stimulating the formation of the NLRP3 inflammasome. Gasdermin D is cleaved *via* caspase-1, leading to pore formation (Coll et al., 2022). In non-canonical signalling, activation of caspase-4/5 stimulates GSDMD cleavage. The resulting amino-terminal fragment (GSDMD-NT) initiates pyroptosis and NLRP3 activation, stimulating release of DAMPs including ATP which can then activate the canonical pathway *via* P2X7R. In the alternative pathway, Toll or interleukin-1 receptor (TIR) domain-containing adaptor inducing interferon-β (TRIFF) and TLR4-receptor-interacting protein kinase 1 (RIPK1) interaction leads to the formation of a RIPK1-Fas-associated with death domain protein (FADD)-pro-caspase-8 complex. Pro-caspase-8 is cleaved, allowing mature caspase-8 to activate the NLRP3 inflammasome, ahead of IL1ß and IL18 excretion.

Efforts to elucidate a common mechanism by which NLRP3 is regulated has led to the classification of three distinct pathways: the canonical (classical), the non-canonical and the alternative pathway (Wang and Hauenstein, 2020), highlighted in Figure 1. Of these, the most thoroughly studied is the canonical pathway, which is activated *via* P2X7R or TLR4 dependent mechanisms, initiating the production of mature IL1β and IL18 *via* caspase-1 mediated cleavage (Song et al., 2021). In addition, caspase-1 mediates the cleavage of gasdermin D (GSDMD), generating an N-terminal cleavage product (GSDMD-NT) which forms lytic pores through which IL1β and IL18 are released. The mechanism of GSDMD cleavage evokes pyroptosis, a highly inflammatory form of regulated cell death (Chen et al., 2021). In addition to this pathway which we traditionally associate with sterile injury, the non-canonical pathway is stimulated by lipopolysaccharide (LPS)-mediated caspase-4/5 activation, which cleaves GSDMD, permitting potassium efflux (Rühl and Broz, 2015; Chen et al., 2021) and caspase-1-mediated production and release of IL1β and IL18. These events are further perpetuated by the release of DAMPs including ATP (Downs et al., 2020), a known activator of the canonical pathway (Downs et al., 2020). Furthermore and distinct from both the canonical and non-canonical pathways, the alternative pathway is specific to monocytes (Mangan et al., 2018) and is independent of potassium efflux (Yu et al., 2021). It relies on a receptor-interacting serine/threonine-protein kinase 1 (RIPK1)-Fas-associated with death domain protein (FADD)-caspase-8 pathway (Chen et al., 2021). In summary and irrespective of the pathway which is activated, increased activation of the NLRP3 inflammasome leads to the regulation and execution of inflammatory responses, a process that becomes dysregulated in diseases including diabetic nephropathy (Shen et al., 2022).

This review will evaluate a role for the priming and activation of the NLRP3 inflammasome in the context of the pathogenesis and progression of diabetic nephropathy. We will summarise current knowledge by describing identified mechanisms within the glomerular and proximal region of the diabetic kidney, before exploring the potential for targeting the NLRP3 inflammasome and its components as a future therapy in NLRP3-induced inflammatory diabetic kidney disease.

### NLRP3 Inflammasome Activation Leads to Glomerular Injury

Our kidneys contain approximately 1 million nephrons which filter blood and create urine (Bertram et al., 2011). The early portion of the nephron contains the glomerulus (Ludwig, 1843), which is a complex of small capillaries, supported by glomerular mesangial cells and surrounded by the Bowman’s capsule (Pollak et al., 2014). The glomerulus is lined by the glomerular basement membrane and podocytes that form foot processes (pedicels), which are sieve-like structures that permit the filtration of water and protein-free soluble substrates into the Bowman’s capsule. The filtrate flows into the renal tubules for selective reabsorption and secretion and the formation of urine (Pollak et al., 2014). Glomerular injury is the initial presentation of diabetic nephropathy (Ilyas et al., 2017), and is characterised by mesangial expansion (Tung et al., 2018), basement membrane thickening (Dongling Liu et al., 2020) and podocyte apoptosis (Weil et al., 2012; Mallipattu and Kravets, 2020). Clinically relevant changes in glomerular disease include proteinuria, albuminuria, albumin/creatinine ratio and glomerular hyperfiltration, all of which are early markers of injury (Treacy et al., 2019). A link between NLRP3 inflammasome activity and glomerular injury in the kidneys of people with diabetic nephropathy is now well established (Shahzad et al., 2015), with evidence that increased glomerular NLRP3 expression (Shahzad et al., 2015; Hou et al., 2020) is paralleled by elevated levels of serum IL1β, the latter of which exhibits a positive correlation to albuminuria (Shahzad et al., 2015).

Several studies have since illustrated the widespread impact of NLRP3 inflammasome activation in diabetic nephropathy through its role in glomerular injury (Xu et al., 2021; Zhan et al., 2020; Shen et al.,2022; Feng et al., 2021). Research by Wu *et al.*, studied the effects of NLRP3 in the kidneys of streptozotocin (STZ)-induced diabetic mice using an NLRP3 knock-out model (Wu et al., 2018). Streptozotocin induces T1DM in mice and rats through the destruction of pancreatic islet ß-cells. The model is widely used for studying T1DM and the complications of diabetes, including the early stages of diabetic nephropathy (Furman, 2021). Wu *et al.*, reported that STZ mice exhibited increased inflammation of their kidneys compared to wild type control, as evidenced by increased expression of monocyte chemoattractant protein 1 (MCP1), increased macrophage infiltration and increased expression of inflammasome markers NLRP3, caspase-1, cleaved IL1β and IL18. With NLRP3 activation linked to increased extracellular matrix deposition and renal fibrosis (Alyaseer et al., 2020), it is unsurprising that these mice reportedly demonstrated increased expression of fibronectin, collagen I and collagen IV (Wu et al., 2018). Knock-out of NLRP3 ameliorated these changes culminating in a reduction in glomerular basement membrane thickening, decreased podocyte foot process effacement, mesangial expansion and glomerular hypertrophy (Wu et al., 2018). Reduced cellular injury led to improvements in physical and biochemical parameters in these mice compared to wild type STZ-induced diabetic mice, including reduced kidney/body weight ratio, albuminuria, urine albumin/creatinine ratio and creatinine clearance (Wu et al., 2018).

Studies utilising both a mouse model of obesity-induced T2DM (db/db) and rat mesangial cells treated with high (30 mM) glucose, reported that podocyte injury, basement membrane thickness, foot process effacement and fibrosis, were all decreased when mice and cells were treated with MCC950, a small molecule inhibitor that blocks NLRP3 activation (Zhang et al., 2019). Similarly, NLRP3 inhibition in db/db mice improved morphological parameters (mesangial expansion, glomerular hypertrophy, glomerulosclerosis and tubulointerstitial damage) and decreased disease biomarkers (serum creatinine, urinary albumin to creatinine ratio), further corroborating the beneficial effects of reducing inflammasome activation in the diabetic kidney (Zhang et al., 2019). In contrast, research by Østergaard *et al.* on T1DM mice failed to demonstrate renoprotective effects of NLRP3 inhibition (Østergaard et al., 2022). In their study, use of MCC950 in STZ-induced diabetic mice identified detrimental effects linked to NLRP3 inhibition, which resulted in upregulation of the expression of markers of inflammation (IL1β, IL18 and MCP1) and fibrosis (collagen I, collagen IV and transforming growth factor beta 1 (TGFβ1)) in addition to increased immune cell infiltration, mesangial expansion, and glomerulosclerosis (Østergaard et al., 2022). These contrary findings highlight the need for further exploration into the effects of NLRP3 inhibition in the kidney.

One of several mechanisms through which NLRP3 drives glomerular disease is through increased pyroptosis (Zhan et al., 2020; Xu et al., 2021), a form of programmed cell death stimulated by inflammation (Lin et al., 2020). Renal tissue from high fat diet (HFD)/STZ-treated mice exhibited mitochondrial swelling, perforation and dissolution of the cell membrane, small vacuole formation on the cell surface and nuclear rupture, characteristics associated with pyroptosis (An et al., 2020). Treatment with punicalagin, a phenolic compound found in pomegranates, reduced expression of inflammasome associated proteins NLRP3, IL1β, caspase-1 and GSDMD. Punicalagin ameliorated a diabetes-induced increase in pyroptosis, and restored markers of renal function including a lowering of the urine albumin to creatinine ratio, decreased glomerular basement membrane thickening, mesangial expansion, and glomerular fibrosis (An et al., 2020). Thioredoxin-interacting protein (TXNIP) interacts directly with NLRP3 and is involved in inflammasome activation and subsequent pyroptosis (Qayyum et al., 2021). Use of lentivirus vector-mediated TXNIP short hairpin RNA (LV-TXNIP-shRNA) to dampen inflammasome activation in HFD/STZ mice *via* TXNIP inhibition reduced pyroptosis mediated cell death (Ke et al., 2020). Reduced inflammasome activity and pyroptosis improved the pathological changes of diabetic nephropathy in the glomerulus including reduced glomerular hypertrophy, basement membrane thickening, mesangial expansion and fibrosis (Ke et al., 2020).

Aside from studies that outline a role for NLRP3 in the induction of pyroptosis, the effect of the inflammasome on podocytes was demonstrated in high (20–40 mM) glucose-treated primary murine podocytes where increased NLRP3 expression impaired podocyte autophagy and exacerbated podocyte damage, effects attenuated using NLRP3 silencing (si) RNA (Hou et al., 2020). Similarly, and as a negative regulator of NLRP3, miRNA miR-10a and 10-b binds to the 3’ untranslated region of NLRP3 messenger (m)RNA and was demonstrated in STZ-induced diabetic mice and human kidney-2 (HK-2) proximal tubule cells to reduce caspase-1 cleavage and IL1β maturation in the diabetic kidney (Ding et al., 2021). This inhibitory role was further supported by studies demonstrating a reduction of miR-10 expression in both kidney biopsy material isolated from individuals with diabetic nephropathy and in STZ-induced diabetic mice (C57BL/6 J), observations paralleled by increased NLRP3 activation (Ding et al., 2021) and corroborated by studies in miR-10 overexpressing STZ-treated mice, where reduced inflammasome activation attenuated an STZ-induced increase in albuminuria, mesangial expansion and inflammation, culminating in improved kidney function (Ding et al., 2021).

Collectively these studies demonstrate the detrimental effects of inflammasome activation in the glomerular region of the diabetic kidney. Targeting NLRP3 significantly reduces physical, biochemical, and molecular markers associated with diabetic nephropathy, providing protection from high glucose-induced glomerular injury, changes which initiate progression of kidney injury in both T1DM and T2DM.

### The Role of NLRP3 Activation in Tubular Inflammation and Fibrosis

Whilst diabetic nephropathy is considered a glomerular disease in origin, it is damage to the proximal tubular region which dictates disease progression. With inflammation and fibrosis of the tubule exhibiting a positive correlation to the rate of entry into end-stage renal disease (ESRD), the therapeutic implications of targeting this damage are irrefutable (Liu et al., 2018). Tubular injury manifests itself in response to increased inflammation (Pérez-Morales et al., 2019), fibrosis (Zeng et al., 2019), immune cell recruitment (Zheng and Zheng, 2016), epithelial-to-mesenchymal transition (EMT) (Sheng and Zhuang, 2020) and extracellular matrix deposition (ECM) (Zhao et al., 2020). Furthermore, increased in renal biopsies of people with diabetic nephropathy, NLRP3 and IL1β expression and activity is also reportedly increased in people with tubulointerstitial injury as compared to those without (Cheng et al., 2021). Several studies describe the role of NLRP3 in driving tubulointerstitial injury in diabetic kidney disease (Ding et al., 2018; Zhang and Wang, 2020).

A major pathology in tubulointerstitial fibrosis is EMT, the trans-differentiation of epithelial cells to myofibroblasts. Characterised by a loss of the epithelial phenotype and the acquisition of pro-fibrotic characteristics, EMT increases fibrosis in the renal tubules driving progression of disease (Sheng and Zhuang., 2020). Human kidney-2 (HK-2) proximal tubule cells when treated with high (30 mM) glucose, exhibit increased NLRP3 inflammasome activation, as demonstrated by increased mRNA and protein expression of NLRP3, ASC, caspase-1, active IL1β and active IL18 (Song et al., 2018). Moreover, NLRP3 inhibition using both short hairpin (sh) RNA and an inflammasome blocking antioxidant N-acetyl-l-cysteine (NAC), negated EMT induced morphology and reversed changes in expression of classical EMT markers alpha-smooth muscle actin (αSMA) and E-cadherin in HK-2 cells (Song et al., 2018). Antioxidant 4-hydroxy-3-methoxyacetophenone (apocynin) was also shown to inhibit NLRP3 inflammasome activity in STZ-treated rats, conferring protection through its ability to reduce glomerular injury and urine creatinine levels, whilst decreasing TGFβ1, fibronectin and collagen expression in the tubules (Xin et al., 2018).

Further *in vitro* studies in HK-2 cells report high (30 mM) glucose-induced NLRP3 inflammasome activation, through class B scavenger receptor CD36 (Hou et al., 2021). Whilst caspase-1 activity drives pyroptosis through GSDMD cleavage, in the absence of GSDMD, caspase-1 can induce apoptosis *via* truncation of the pro-apoptotic protein BH3 Interacting Domain Death Agonist (Bid), culminating in activation of caspase-9 and increased caspase-3 activity (Tsuchiya et al., 2019; Heilig et al., 2020). In HK-2 cells, inflammasome activation initiated caspase-3 mediated apoptosis, and a shift in the expression of B-cell lymphoma protein 2 associated X (Bax) and B-cell lymphoma protein 2 (BCL2) protein, referred to as the Bax/BCL-2 ratio. In healthy cells the pro-apoptotic properties of Bax are counteracted by the apoptosis inhibiting effects of BCL-2, however, when this balance is disturbed to favour the expression of Bax, cells become apoptotic. These inflammation-induced changes in markers of apoptosis were negated by NLRP3 shRNA (Hou et al., 2021). As a form of programmed cell death initiated when a cell is damaged beyond repair, apoptosis is a widespread marker of renal injury (Priante et al., 2019). In db/db mice, CD36 knockdown reduced inflammasome activation which conferred protection *via* reduction of apoptosis, macrophage infiltration and proximal tubule injury (Hou et al., 2021). The protective effects of NLRP3 inhibition were similarly observed in high glucose treated rat proximal renal tubular epithelial cells (NRK-52E cells), whereas with their observations in glomeruli of the diabetic kidney, Ke *et al* report that LV-TXNIP-shRNA transfection significantly reduced apoptosis in response to downregulated expression of NLRP3, ASC, cleaved caspase-1, GSDMD-NT, IL1β and IL18 (Ke et al., 2020).

In summary, these findings support the concept that NLRP3 inflammasome activation is instrumental in mediating renal tubule damage in diabetic nephropathy. The benefits of targeting inflammasome activity, as shown in these studies, provides hope for a therapeutic and preventative measure to significantly reduce the number of people who develop diabetes driven ESRD.

### Therapeutic Approaches to Targeting NLRP3 Inflammasome Activity

Despite its role in disease progression, treatment of inflammation in the kidney represents an unmet clinical need. Whilst early stages of diabetic nephropathy are managed *via* control of blood glucose and pressure (Selby and Taal, 2020), many people still progress to late-stage disease despite good glycaemic control, thus limiting treatment options to dialysis and transplantation, both of which negatively impact quality of life (Selby and Taal, 2020). Sodium-glucose co-transporter 2 inhibitors (SGLT2i) are the most recent and promising development in the treatment of diabetes and have recently been approved for use in diabetic nephropathy for people with T2DM. The inhibitors block glucose reabsorption in the proximal tubule, increasing glucose excretion in the urine, allowing blood glucose to be controlled without the risk of hypoglycaemia (Shaffner et al., 2021), reducing glomerular hyperfiltration through sodium mediated activation of tubuloglomerular feedback (Davidson, 2019). Dapagliflozin, an SGLT2 inhibitor, has been shown to reduce renal injury even in the absence of diabetes, through inhibition of the NLRP3 inflammasome, protecting against kidney fibrosis (Ke et al., 2022). Unfortunately, whilst SGLT2i represent a major advance in the treatment of diabetic nephropathy, limitations of wide range suitability and potential side effects including diabetic ketoacidosis (Douros et al., 2020), genital infections (Liu et al., 2017) and lower limb amputation (Neal et al., 2017) suggest that they are not a one size fits all, and so other options must also be explored (Nelinson et al., 2021).

Due to the prominent role that the NLRP3 inflammasome plays in a plethora of chronic inflammatory diseases (Seok et al., 2021), it is an attractive target for therapeutic interventions (Coll et al., 2022). Many biological elements are essential for inflammasome activation, and this provides several opportunities for inflammasome inhibition, including inhibition of transcription factors (NFκB), inflammasome components (NLRP3, ASC, caspase-1), and downstream inflammasome mediators (IL1β). Whilst no NLRP3 blockers are clinically available, several inhibitors are currently in trials. Dapansutrile (OLT1177), an NLRP3 specific inhibitor, is currently in phase II clinical trials for the treatment of Covid-19 induced cytokine storm (Clinical Trial identifier: NCT04540120, Olatec Therapeutics, 2022) and gout flares (Klück et al., 2020). The pharmaceutical company Inflazome (Roche) has developed two oral NLRP3 inhibitors, Inzomelid (NCT04086602) and Somalix, both of which have completed Phase 1 trials and have been tested in people with Cryopyrin-associated periodic syndrome (CAPS) (Coll et al., 2019). Another NLRP3 inhibitor that has completed Phase I clinical trials in healthy individuals is ZYIL1 (NCT04731324) which is now progressing onto Phase II clinical trials in people with CAPS syndrome (Clinical Trials Arena, 2021). The compound IFM-2427, recently renamed DFV890, is a systemic NLRP3 antagonist which has recently completed Phase I and Phase II clinical trials for several inflammatory conditions (CDFV890D12201, NCT04382053, CDFV890B12201, CDFV890A12201, NCT04382053). Despite early promise, no mechanism of action has so far been reported, therefore its potential use in diabetic nephropathy is unknown.

Whilst results from these trials appear promising, outcomes have been met with concerns regarding suppression of the innate immune response, where NLRP3 activation is required in the face of microbial infection. Efforts to dampen DAMP induced sterile inflammation have to date proven unsuccessful, with attempts to block the human P2X7 receptor producing variable pharmacodynamic responses, perhaps as a consequence of genetic variability (McHugh et al., 2012; Burnstock and Knight, 2017). On the other hand, targeting downstream mediator IL1β using recombinant human monoclonal antibody ACZ885 (Canakinumab) yielded disappointing results in trials in T1DM (NCT00947427) and diabetic retinopathy (NCT01589029), with increased rates of infection and sepsis in the face of a dampened inflammatory response (Ridker et al., 2017). The ineffectiveness of IL1β blockers could also be due to alternative inflammasome pathways remaining active (Moran et al., 2013), such as IL18 release (Hirooka and Nozaki, 2021) and pyroptosis (Lin et al., 2020). A consensus appears to be that these drugs would be more effectively used in combination with other anti-inflammatory drugs, however, this raises new safety considerations in relation to side effects, potential and harmful drug interactions and toxicity of the liver (Sriuttha et al., 2018). Consequently, further research is needed to identify clinically safe and effective therapeutics for NLRP3 driven chronic inflammatory diseases, including diabetic nephropathy.

## Conclusion

Globally, 537 million adults are currently living with diabetes and its secondary complications, including diabetic nephropathy (International diabetes federation diabetes Atlas, 2021) Associated with a range of chronic inflammatory diseases, including atherosclerosis (Sharma et al., 2021), diabetes (Ding et al., 2019) and obesity (Qin et al., 2021), recent research highlights the key role of NLRP3 in both glomerular (Shahzad et al., 2015; Ilyas et al., 2017; Hou et al., 2020) and tubular injury (Ding et al., 2018; Zhang and Wang, 2020) in the context of diabetic nephropathy. The NLRP3 inflammasome can initiate immune cell recruitment (Niu et al., 2019) and resulting inflammation (Yang et al., 2019), downstream EMT (Song et al., 2018), pyroptosis (Zhang and Wang, 2020) and apoptosis (Dai et al., 2019), leading to fibrosis (Li et al., 2019). Specifically, NLRP3 activation drives glomerular disease through pyroptosis (Ke et al., 2020), leading to mesangial expansion (Tung et al., 2018) and glomerular thickening (Yanfei Liu et al., 2020). Late-stage nephropathy includes damage to the proximal tubule region of the kidney, where increased damage is positively correlated with disease progression (Takaori et al., 2016). Interestingly, whilst the role of the NLRP3 inflammasome in kidney disease pathogenesis is irrefutable, there is a clear demand for increased studies to outline the exact nature of the pathway involved. A clearer understanding will undoubtedly unveil future therapeutic targets.

Although targeting the NLRP3 inflammasome has to date been shown to be effective in slowing disease progression and onset, there are currently no approved treatments which can ablate and/or downregulate its activity, a consequence of side effects and increased susceptibility to infection (Niu et al., 2019). Exploring novel therapeutics alongside further research into the specific targeting of NLRP3 inflammasome components will undeniably offer hope for treating NLRP3-inflammasome associated diseases, such as diabetic nephropathy (Kim et al., 2015; Sha et al., 2017; Mugisho et al., 2018; International Diabetes Federation, 2019; Zhao et al., 2019; Sun et al., 2020; Wang et al., 2020; Al Mamun et al., 2021; Samsu, 2021; ClinicalTrials.gov, 2022).
